# The impact of inspiratory muscle training on pulmonary function recovery and pulmonary complications in patients undergoing cardiothoracic surgery: a systematic review and meta-analysis

**DOI:** 10.3389/fphys.2026.1761926

**Published:** 2026-04-21

**Authors:** Yinping Ge, Min Zhang, Yun Gan

**Affiliations:** 1 Department of Thoracic Surgery, Linping Campus, The Second Affiliated Hospital of Zhejiang University School of Medicine, Hangzhou, China; 2 Department of Neurosurgery, The Second Affiliated Hospital, Zhejiang University School of Medicine, Hangzhou, China; 3 Department of Infection Control, The Second Affiliated Hospital, Zhejiang University School of Medicine, Hangzhou, China

**Keywords:** cardiothoracic surgery, inspiratory muscle training, meta-analysis, postoperative pulmonary complications, pulmonary function

## Abstract

**Objective:**

To quantify the effects of inspiratory muscle training (IMT) on pulmonary function (PF) recovery and postoperative pulmonary complications (PPCs) including pneumonia and atelectasis in patients undergoing cardiothoracic surgery through systematic evaluation and meta-analysis, providing an evidence-based basis for perioperative respiratory management.

**Methods:**

PubMed, Embase, Cochrane Library, and Web of Science were systematically searched until May 2025, with 7 randomized controlled trials (RCTs) involving 507 adult patients who underwent cardiothoracic surgery retrieved (the study of transcatheter aortic-valve replacement was excluded due to heterogeneous surgical characteristics). The quality of the literature was evaluated using the Cochrane Risk-of-Bias Tool (RoB 2.0). A meta-analysis was conducted using RevMan 5.4 software to compare the differences in predefined outcome measures: forced expiratory volume in 1 second (FEV1), forced vital capacity (FVC), and the pneumonia incidence between the IMT and control groups.

**Results:**

The meta-analysis showed that the IMT group had a higher FEV1 than the control group [mean difference (MD)=0.80 L, 95% confidence interval (CI): 0.09-1.52, P = 0.03], with clinically relevant improvements. Similarly, FVC was better in the IMT group (MD = 0.64 L, 95% CI: 0.11-1.17, P = 0.03), also representing a clinically meaningful benefit. However, there was no difference in FEV1/FVC ratio between the two groups (P = 0.15). The IMT group performed better in the 6-minute walk test (6MWT) (MD = 47.89 m, 95% CI: 1.28-94.51, P = 0.04), indicating improved functional capacity. Regarding PPCs, the incidence of postoperative pneumonia [odds ratio (OR)=0.18, 95% CI: 0.06-0.57, P = 0.004] and atelectasis (OR = 0.37, 95% CI: 0.17-0.81, P = 0.01) in the IMT group were lower than those in the control group.

**Conclusion:**

IMT can effectively improve PF and reduce the risk of PPCs in patients undergoing cardiothoracic surgery by enhancing the strength and endurance of inspiratory muscles.

## Introduction

Cardiothoracic surgery is often accompanied by respiratory muscle dysfunction, decreased lung volume, and complications such as lung infection and atelectasis, which seriously affect the rehabilitation process and quality of life (Ehrsam and Aigner, 2023). Statistics indicate that an overall incidence of about 20% to 40% of patients undergoing cardiothoracic surgery develop postoperative pulmonary complications (PPCs), with rates varying significantly depending on the surgical approach (e.g., open vs. minimally invasive) and patient risk profiles (Lin et al., 2022; Litle, 2023). Notably, different subtypes of cardiothoracic surgery have significant differences in the degree of respiratory impairment: open thoracic/cardiac surgery with thoracic incision is accompanied by severe respiratory muscle dysfunction and lung volume reduction, while minimally invasive cardiac procedures such as TAVR involve no thoracic incision, no one-lung ventilation and minimal surgical trauma, leading to slight and transient respiratory drive impairment. Respiratory muscle dysfunction—characterized by reduced strength and endurance of the diaphragm and external intercostals—plays a key role in the pathogenesis of these complications, as it impairs lung ventilation, promotes alveolar collapse, and increases the risk of mucus retention. Therefore, exploring safe and effective perioperative interventions to improve pulmonary function (PF) and reduce the risk of complications has become a key issue in cardiothoracic surgery nursing.

Inspiratory muscle training (IMT), a rehabilitation technique, enhances the strength and endurance of inspiratory muscles (the diaphragm, external intercostals, etc.) through targeted resistance exercises (Azambuja et al., 2020). Prolonged disuse of inspiratory muscles due to postoperative immobilization, chronic diseases, etc., or pathological conditions like neuromuscular injuries, can lead to a decline in muscle strength (McNarry et al., 2022). The training is typically performed using devices with controllable inspiratory resistance (e.g., threshold load devices, spring resistance devices) and can be initiated preoperatively, postoperatively, or both. The standard protocol usually includes 10–20 minutes of training per session, 5–7 times per week, with progressive adjustment of resistance based on individual tolerance (Bissett et al., 2023; Polastri et al., 2024). It is commonly administered by rehabilitation therapists or nurses, with regular monitoring of training compliance and progress. In recent years, IMT has gained consistent recognition in clinical practice for improving respiratory function in surgical patients (da Silva Guimaraes et al., 2021; Mello et al., 2024). However, current clinical application research on IMT still remains controversial. For example, the research results of Fernández-Lázaro D indicate that inconsistencies in IMT training intensity, duration, and initiation timing may lead to variations in outcomes (Fernandez-Lazaro et al., 2021), while Sakai Y et al. state that IMT has no effect on patients undergoing lung cancer surgery (Sakai et al., 2023). These discrepancies can be categorized by four key factors: (1) training timing and duration (e.g., preoperative vs. postoperative initiation, short-term vs. long-term training cycles); (2) training dosage (inconsistent resistance loads across studies); and (3) patient population differences. For example, Sakai Y et al. reported no effect of IMT in patients undergoing lung cancer surgery, which may be attributed to suboptimal training dosage and late initiation timing; and (4) routine nursing variability among centers—different medical centers have different conventional perioperative respiratory care measures, and individual centers integrate low-intensity breathing exercises into routine nursing, which may reduce the efficacy difference between the IMT group and the control group.

Given the inconsistent evidence and unaddressed heterogeneity in existing studies—especially the high heterogeneity of surgical subtypes in cardiothoracic surgery (e.g., transcatheter aortic-valve replacement with minimal thoracic trauma vs. open thoracic/cardiac surgery with severe respiratory impairment)—this systematic review and meta-analysis aimed to quantify the impact of IMT on primary outcomes (pulmonary function [PF] parameters including forced expiratory volume in 1 second (FEV1) and forced vital capacity (FVC), and incidence of PPCs including pneumonia and atelectasis) in patients undergoing cardiothoracic surgery (the study of transcatheter aortic-valve replacement was excluded due to heterogeneous surgical characteristics). The findings are expected to clarify the actual value of IMT in cardiothoracic surgery with significant thoracic/cardiac trauma, provide evidence-based support for the development of standardized perioperative respiratory muscle training programs, and inform the integration of IMT into Enhanced Recovery After Surgery (ERAS) strategies for cardiothoracic surgery.

## Materials and methods

### Literature retrieval strategy

Database selection: A systematic search was conducted in four core literature databases to ensure comprehensive coverage of relevant studies. The selection rationale for each database is as follows: (1) PubMed: Covers a wide range of clinical randomized controlled trials (RCTs) and high-impact studies in respiratory medicine, surgery, and rehabilitation; (2) Embase: Provides extensive coverage of rehabilitation, perioperative care, and thoracic surgery literature, with robust indexing of non-English studies; (3) Cochrane Library: Focuses on systematic reviews and RCTs, offering high-quality evidence in clinical practice; (4) Web of Science Core Collection: Includes interdisciplinary research, ensuring no omission of relevant studies in surgery and rehabilitation. The search period spanned from database inception to May 2025.

Search terms: A combination of controlled vocabularies and free-text terms was used to optimize retrieval sensitivity. Specific controlled vocabularies included: Medical Subject Headings (MeSH) in PubMed, Emtree terms in Embase, and Cochrane Controlled Trials Register (CCTR) terms in Cochrane Library. Free-text terms were consistent across databases. Search terms were clearly grouped and combined using Boolean logic (AND/OR), with consistent quotation marks for multi-word terms. The complete search strategy is as follows: (“Inspiratory Muscle Training” OR “IMT” OR “respiratory muscle training”) AND (“cardiothoracic surgery” OR “lung cancer surgery” OR “esophageal surgery”) AND (“pulmonary function” OR “postoperative pulmonary complications” OR “pneumonia” OR “atelectasis”).

This study followed the Preferred Reporting Items for Systematic Reviews and Meta-Analyzes (PRISMA) statement and was registered on the PROSPERO platform (Registration number: CRD420261337035). The inclusion of “lung cancer surgery” and “esophageal surgery” as search terms reflects the mixed cardiothoracic surgery population of interest, which encompasses pulmonary, esophageal, and cardiac-related intrathoracic surgeries as defined in the inclusion criteria.

### Inclusion and exclusion criteria

Inclusion criteria: (1) Randomized controlled trials (RCTs); (2) Adult patients (≥18 years old) undergoing cardiothoracic surgery, including thoracic surgery (e.g., thoracoscopic lobectomy), esophageal surgery (e.g., esophagectomy), and cardiac surgery (TAVR studies were excluded from final analysis due to heterogeneous surgical characteristics); (3) The experimental group received IMT (preoperative, postoperative, or perioperative training), while the control group received routine nursing, sham training (e.g., non-resistance breathing exercises), or no intervention. Routine nursing was defined as standard perioperative care including respiratory health education, posture guidance, pain management, and general postoperative care without targeted inspiratory muscle training; (4) Outcome measures (reporting at least one of the following measures): (i) PF indicators (forced vital capacity [FVC], forced expiratory volume in one second [FEV_1_], maximal inspiratory pressure [MIP], total lung capacity [TLC], etc.); (ii) Incidence of postoperative pulmonary complications (PPCs) including pneumonia and atelectasis were defined based on the criteria reported in each included study: pneumonia was diagnosed by a combination of clinical symptoms (fever, cough, purulent sputum), laboratory tests (elevated leukocyte count, positive sputum culture), and radiological findings (pulmonary infiltrates); atelectasis was confirmed by chest X-ray or computed tomography showing collapsed lung tissue. Due to slight variations in diagnostic criteria across studies, heterogeneity was assessed and discussed. Exclusion criteria: (1) Studies that are duplicates or have overlapping data; (2) Studies without a clear IMT protocol (e.g., resistance load, training cycle) or incomplete outcome data; (3) Non-peer-reviewed gray literature (including conference abstracts, unregistered trial data, and unpublished theses) or data that cannot be obtained. The rationale for excluding gray literature is that such studies often lack complete data reporting (e.g., detailed IMT implementation protocols, outcome measurement procedures, and statistical analysis methods) and cannot be rigorously evaluated using the Cochrane Risk-of-Bias Tool (RoB 2.0). Incomplete or unvalidated data may introduce selection bias and reduce the reliability of the meta-analysis results.

### Literature screening and data extraction

Two researchers with prior training in systematic review methodology (in line with PRISMA guidelines) and experience in thoracic surgery and rehabilitation research independently read the titles and abstracts for initial screening, excluding studies that were obviously irrelevant. For the remaining literature, full texts were obtained for review, and a cross-check mechanism was established (Endnote X8.3 software). Disagreements were first resolved through in-depth discussion; if consensus could not be reached after two rounds of consultation, a third reviewer with senior expertise in systematic reviews and meta-analyses was invited for arbitration. A standardized extraction form was designed, and the two researchers independently extracted the following information: author(s), year of publication, sample size, type of surgery, characteristics of study participants, interventions, and outcome measures.

### Literature quality evaluation

The Cochrane Risk-of-Bias Tool 2.0 (RoB 2.0) was used to evaluate the methodological quality of included randomized controlled trials (RCTs), in strict accordance with the tool’s standardized framework.

Assessment domains were aligned with RoB 2.0’s five core domains focusing on bias sources: (1) Bias arising from the randomization process; (2) Bias due to deviations from the intended interventions; (3) Bias due to missing outcome data; (4) Bias in measurement of the outcome; (5) Bias in selection of the reported result.

Consistent with RoB 2.0 requirements, the risk-of-bias assessment was conducted at the outcome level rather than the study level. Specific outcomes evaluated separately included: (1) Pulmonary function parameters (FEV1, FVC, FEV1/FVC); (2) 6 minute walk test [6MWT, all test results were converted to meters (m)]; (3) Postoperative pulmonary complications (PPCs: pneumonia, atelectasis).

Two researchers independently performed the risk-of-bias assessment, and this process was conducted separately from data extraction to ensure methodological independence and minimize potential bias. Each outcome was categorized as “low risk”, “high risk”, or “some concerns” for each domain. Disagreements between the two researchers were resolved through in-depth discussion; if consensus could not be reached, a third researcher with expertise in systematic review methodology was consulted for arbitration.

### Statistical methods

Continuous variables (PF indicators and 6MWT) were analyzed using mean differences (MD) or standardized mean differences (SMD) with 95% confidence intervals (CI), based on consistent criteria: MD was used when outcomes were measured using the same scale across studies (e.g., FEV1 and FVC reported in liters [L] by most studies). SMD was applied when different measurement scales were used for the same outcome (e.g., FEV1 reported in percentage of predicted value [%] by one study and L by others) to standardize results for cross-study comparison. Dichotomous variables (incidence of postoperative pulmonary complications [PPCs], including pneumonia and atelectasis) were evaluated using Odds Ratio (OR) with 95% CI as the preferred measure. Heterogeneity among studies was assessed using two complementary methods: Q test (P < 0.1 indicating statistical heterogeneity). I^2^ statistic, interpreted per Cochrane guidance with nuanced categories: 0-40% might not be important; 30-60% may represent moderate heterogeneity; 50-90% may represent substantial heterogeneity; 75-100% considerable heterogeneity. Overlapping ranges were resolved by integrating Q test results and clinical/methodological context. Model selection (fixed-effect vs. random-effects) was determined by synthesizing statistical, clinical, and methodological heterogeneity: A fixed-effect model was applied when low statistical heterogeneity (I^2^ ≤ 50%) was observed, and no significant clinical (e.g., consistent IMT protocols, homogeneous patient populations) or methodological (e.g., similar blinding strategies, follow-up durations) heterogeneity existed. A random-effects model was used if: (1) substantial/considerable statistical heterogeneity (I^2^ > 50%) was present; or (2) low statistical heterogeneity but obvious clinical/methodological heterogeneity (e.g., mixed surgical subtypes, variable training initiation timing) was identified, to account for between-study variability. Publication bias assessment followed standard methodological criteria: Funnel plots were conducted, and funnel plot analysis suggested low publication bias. Meta-analysis was performed using RevMan 5.4 (Cochrane Collaboration), with Stata 17.0 used to assist in calculating SMD and generating funnel plots for eligible outcomes.

## Results

### Literature screening results

**Figure 1:**
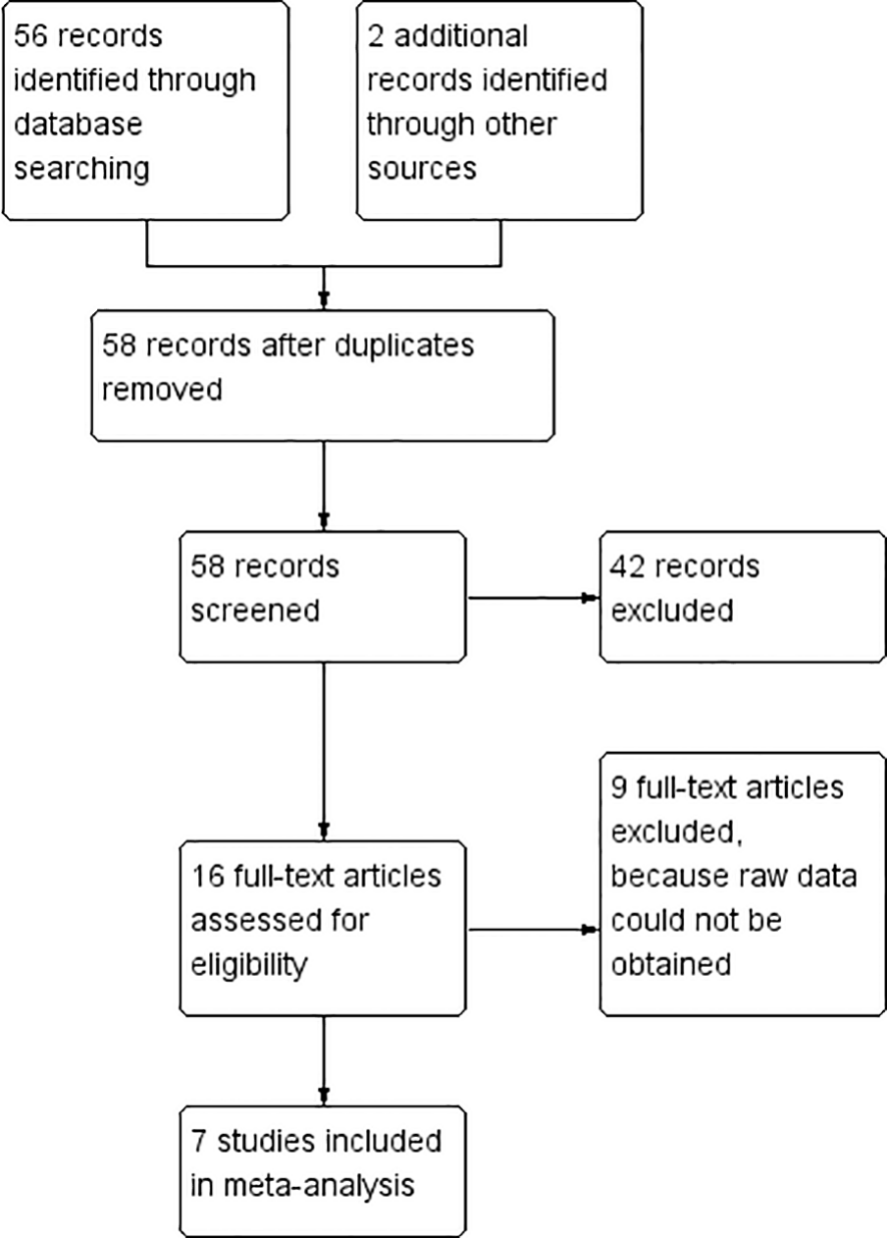
Screening process of literature. Records identified: A total of 56 records were retrieved through systematic searches of PubMed, Embase, Cochrane Library, and Web of Science Core Collection; an additional 2 records were identified through other sources (e.g., reference lists of included studies), resulting in 58 initial records.

After screening, 7 eligible studies were ultimately included (Hegazy et al., 2021; Liu et al., 2021; Zhou and Sun, 2022; Xu et al., 2023; Zhang et al., 2023; Tao et al., 2024; Kulchanarat et al., 2025; Mizusawa et al., 2025) (**Figure 1**). A total of 507 participants were studied across the 7 included RCTs, with 255 patients in the IMT group and 252 in the control group. Except for the research by Hegazy FA et al (Hegazy et al., 2021), the participants of all other studies were over 50 years old (**Table 1**). The included evidence consists of geographically diverse randomized controlled trials (RCTs) from multiple regions (e.g., Asia, Europe, Africa), with consistent RCT design but variations in IMT implementation timing (preoperative, postoperative, or perioperative) and surgical subtypes (pulmonary, esophageal, and cardiac-related cardiothoracic surgery). This age distribution indicates that the study results are more generalizable to middle-aged and elderly patients undergoing cardiothoracic surgery, while caution should be exercised when extending the conclusions to younger surgical populations—given potential differences in baseline respiratory muscle function, postoperative recovery dynamics, and response to IMT between younger and older adults.

**Table 1:** Baseline data of the literature.

Author	Type of surgery	IMT group			Control group			Outcome measures
		Age	Male	n	Age	Male	n	
Hegazy et al., 2021	Mitral valve replacement	39 ± 4.28	25	50	38.3 ± 3.29	26	50	(1)(2)(3)(4)
Kulchanarat et al., 2025	Coronary artery bypass grafting	61 ± 8.61	9	29	64.83 ± 8.08	13	29	(4)(5)(6)
Liu et al., 2021	Video-assisted thoracoscopic surgery	64.2 ± 5.9	12	32	66.3 ± 7.9	10	31	(4)(5)(6)
Mizusawa et al., 2025	Esophagectomy	69.9 ± 4.3	9	15	68.5 ± 8.7	13	17	(1)(2)(3)(4)(5)(6)
Tao et al., 2024	Thoracoscopic lobectomy	58.18 ± 7.05	28	44	57.22 ± 6.14	25	44	(1)(2)(4)
Zhang et al., 2023	Cardiac surgery	55.02 ± 14.40	26	40	56.85 ± 12.56	26	40	(1)(2)(6)
Zhou and Sun, 2022	Thoracoscopic lobectomy	60.3 ± 10.1	26	44	63.2 ± 9.2	23	42	(1)(2)(3)(4)(5)(6)

Outcome measures included (1) FEV1, (2) FVC, (3) FEV1/FVC, (4) 6MWT, (5) incidence of postoperative pneumonia, and (6) incidence of postoperative atelectasis.

Duplicates removed: No duplicate records were found after cross-database verification, retaining 58 records for further screening. Records screened: Titles and abstracts of the 58 records were independently reviewed by two researchers. A total of 42 records were excluded for obvious irrelevance (e.g., non-RCT design, non-cardiothoracic surgery population, no IMT intervention). Full-text articles assessed for eligibility: The remaining 16 records were retrieved as full texts for detailed evaluation. Among these, 9 studies were excluded because raw data could not be obtained or outcome measures did not meet the predefined criteria. Studies included in meta-analysis: Finally, 7 eligible RCTs were included in the meta-analysis.

### Literature quality evaluation

**Figure 2:**
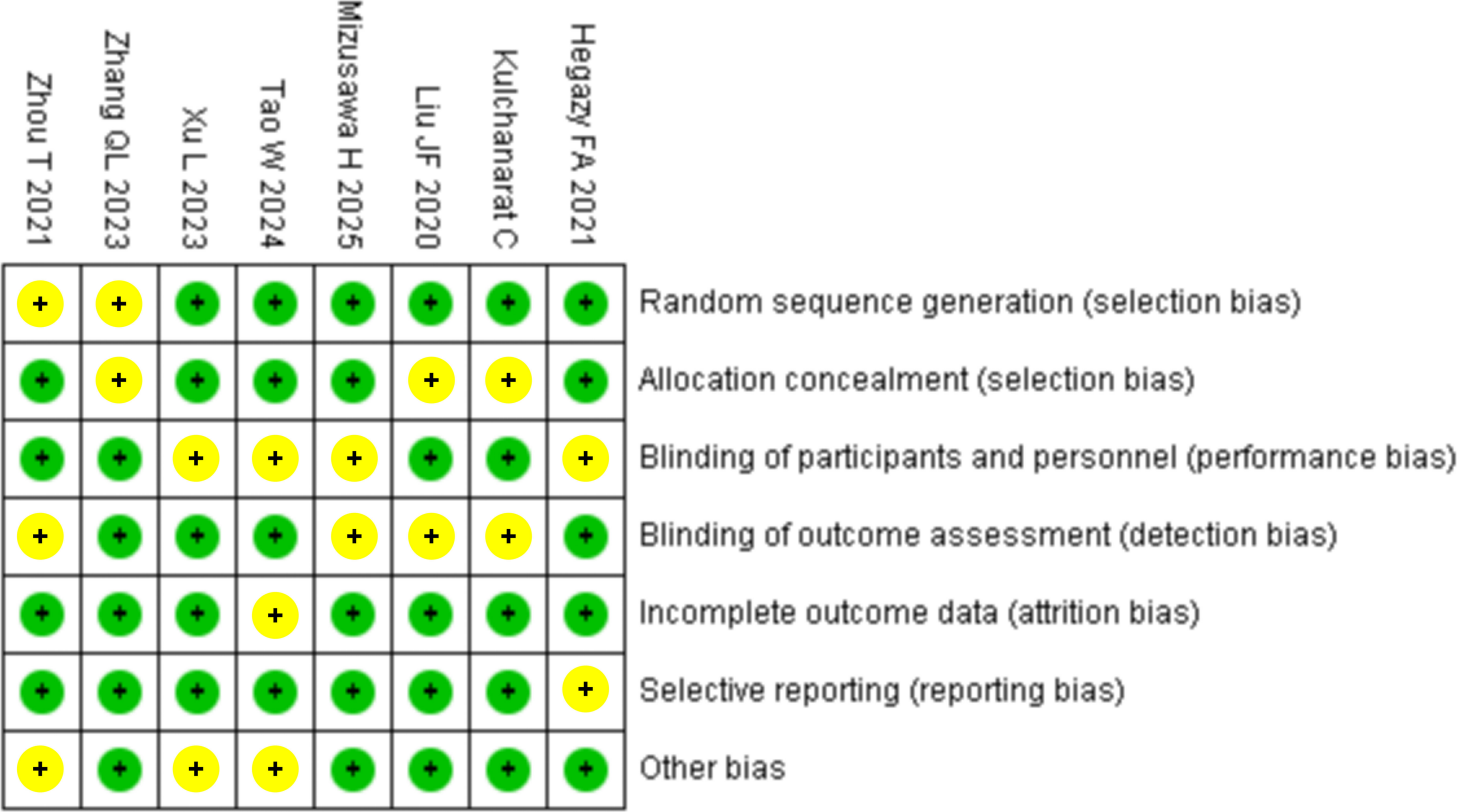
Quality assessment of the literature. Green = low risk; Yellow = some concerns; Red = high risk. The overall risk level of the literature was low risk.

As shown in **Figure 2**, all the studies were of moderate or low risk, with no high-risk literature, confirming that the literature included in this study is of high quality and provides sufficient references.

### Meta-analysis results

#### Analysis of FEV1

**Figure 3:**
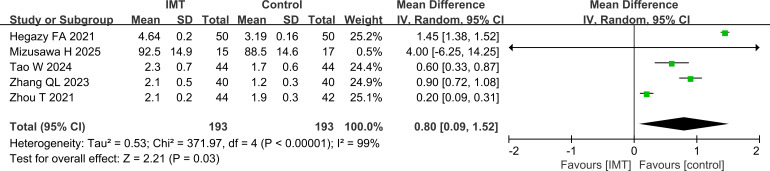
Comparison of FEV1, higher in the IMT group than in the control group.

The results of FEV1 in different literatures were analyzed. Among them, the data units of Mizusawa H et al Mizusawa et al. (2025). were reported as (%), and all others were reported as (L). There was heterogeneity among the articles, and analysis using a random effects model showed that FEV1 was higher in the IMT group than in the control group (P = 0.03) (**Figure 3**).

#### Analysis of FVC

**Figure 4:**
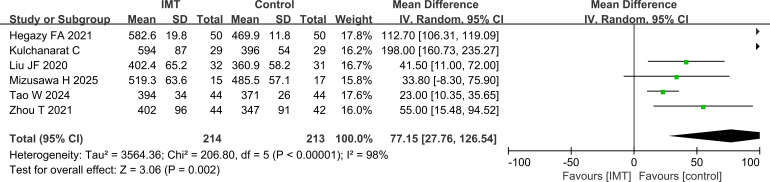
Comparison of FVC, which was higher in the IMT group than in the control group.

Subsequently, the results of FVC were compared. There was also heterogeneity among the studies, and the analysis showed that FVC was also higher in the IMT group than in the control group (P = 0.02) (**Figure 4**).

#### Analysis of FEV1/FVC

**Figure 5:**
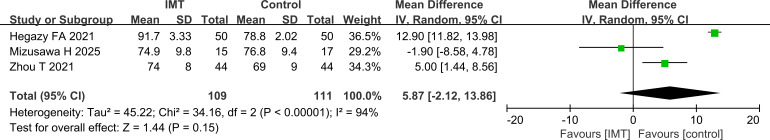
Comparison of FEV1/FVC, no difference between the two groups.

For FEV1/FVC comparison, the random-effects model showed no difference in FEV1/FVC between the IMT group and the control group (P = 0.15) (**Figure 5**).

#### Analysis of the 6MWT

**Figure 6:**
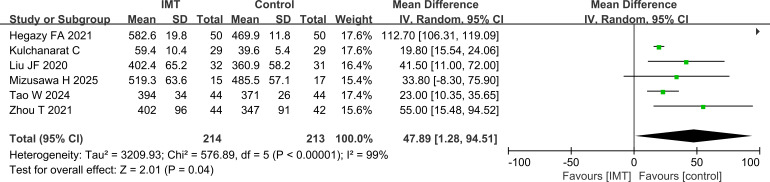
Comparison of the 6MWT, the IMT group was higher than the control group.

In the comparison of 6MWT findings, 6MWT was also higher in the IMT group than in the control group (P = 0.04) (**Figure 6**).

#### Analysis of postoperative pneumonia

**Figure 7:**
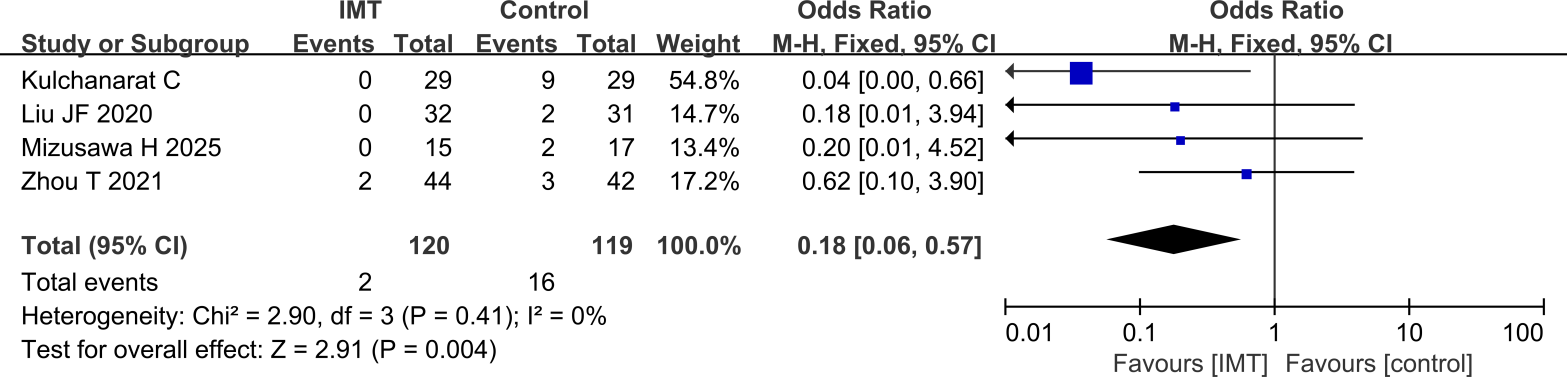
Comparison of incidence of postoperative pneumonia, which was lower in the IMT group than in the control group.

In terms of postoperative complications, 4 articles reported the incidence of postoperative pneumonia. There was no heterogeneity among the studies, and the results of the fixed effect model analysis showed that the incidence of postoperative pneumonia was lower in the IMT group than in the control group (P = 0.004) (**Figure 7**).

#### Postoperative atelectasis analysis

**Figure 8:**
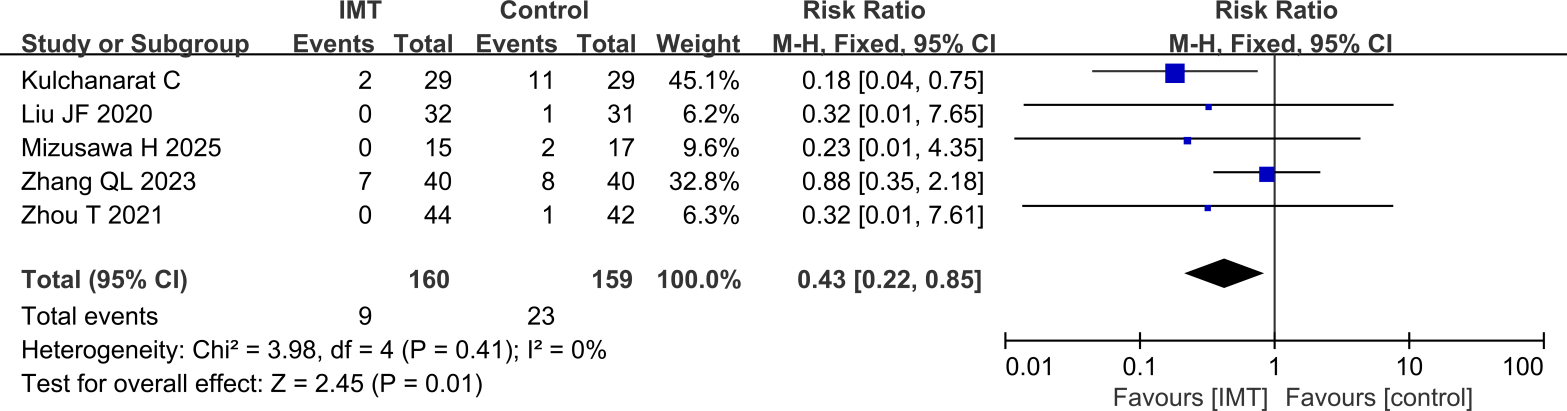
Comparison of the incidence of postoperative atelectasis, lower in the IMT group than in the control group.

Finally, the statistical results of the incidence of postoperative atelectasis in each literature were compared. The incidence of atelectasis was also lower in the IMT group than in the control group (P = 0.01) (**Figure 8**).

#### Publication bias analysis

**Figure 9:**
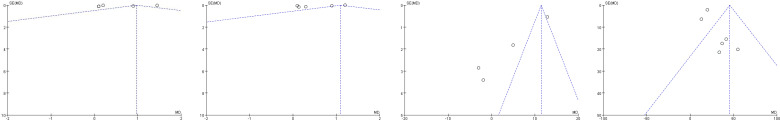
Funnel plot of FEV1, FVC, FEV1/FVC, and 6MWT.

In the analysis of FEV1, FVC, FEV1/FVC, and 6MWT, there was heterogeneity among the literatures, so funnel plots were drawn for these indicators. The results showed that the funnel plots of FEV1 and FVC were basically symmetrical, but the funnel plots of FEV1/FVC and 6MWT were significantly shifted to the left. Through one by one analysis, we found that the bias of the funnel plot disappeared after excluding the literature of Hegazy FA et al., confirming that this literature was the main source of heterogeneity (presumably due to the large difference in the age of the subjects in this literature and other literatures). However, after excluding the literature of Hegazy FA et al (Hegazy et al., 2021), the analysis results of FEV1/FVC and 6MWT were still consistent with the above situation (**Figure 9**).

## Discussion

Although the ERAS concept emphasizes the importance of perioperative respiratory management, there is currently no unified standard for nursing intervention programs for cardiothoracic surgery in clinical practice, and significant inter-center variability in routine perioperative care has been observed across included studies: some centers only provided basic respiratory health education as routine care, while others integrated low-intensity breathing exercises into conventional nursing, which may lead to mild heterogeneity in the control group interventions. This study included 7 RCTs involving 507 patients with cardiothoracic surgery (thoracic resection, esophageal surgery, open cardiac surgery; TAVR excluded), and the results showed that compared with the control group, the IMT group had significantly higher FEV1, FVC and 6MWT, and lower incidence of postoperative pneumonia and atelectasis, suggesting that IMT can improve pulmonary function and postoperative safety in patients with cardiothoracic surgery with significant thoracic trauma/intrathoracic manipulation. Regarding blinding: (1) Blinding of participants and personnel was achieved in 4 studies using sham training devices (e.g., non-resistance devices with identical appearance to IMT devices), which minimized performance bias; (2) Blinding of outcome assessment was not achieved in 3 studies, but the primary outcomes (FEV1, FVC, 6MWT, PPC incidence) were all objective indicators with no subjective judgment bias, and the inter-center variability of routine care had no significant impact on the objective outcome measurement. The lack of full blinding may have a minor impact on results, but the consistency of objective indicators reduces this bias.

Our findings suggest that IMT enhances the strength and endurance of the inspiratory muscles, mainly including the diaphragm and external intercostal muscles, through specific resistance training. The mechanism of action can be analyzed from both physiological and pathological perspectives. On the one hand, IMT acts directly on the inspiratory muscle groups through progressive resistance training, triggering adaptive changes in the muscles. On the other hand, alveolar collapse and pleural effusion following cardiothoracic surgery can easily lead to restrictive ventilatory disorders (Sorino et al., 2022). IMT promotes the reopening of collapsed alveoli and improves the ventilation-perfusion ratio by increasing transpulmonary pressure (i.e., the difference between alveolar pressure and intrapleural pressure) (Van Hollebeke et al., 2025). In an animal experiment, researchers found that IMT may increase the perfusion of collapsed lung tissue and improve the oxygenation index (Jaenisch et al., 2025). This mechanism is closely related to the improvement of FVC in the IMT group in this study and to the reduced incidence of postoperative atelectasis. In addition, IMT has been reported to inhibit the release of pro-inflammatory factors (e.g., IL-6, TNF-α) by regulating the balance of the autonomic nervous system, thereby reducing postoperative lung inflammation (Saka et al., 2021). Moreover, IMT promotes venous return and improves cardiac output by increasing intrathoracic negative pressure (Del Corral et al., 2023). This directly reduces the risk of postoperative pneumonia and helps alleviate surgical incision stress, facilitating recovery. Benli RK et al. further confirmed that IMT indirectly enhances expiratory flow by increasing inspiratory muscle strength, contributing to enhanced mucociliary clearance efficiency (Benli et al., 2024). These benefits can help increase maximal expiratory pressure (MEP) and peak cough flow, thus reducing the risk of mucus retention (Yu et al., 2025). The significantly reduced incidence of atelectasis in the IMT group in this study may be related to a synergistic effect of this mechanism. However, heterogeneity in the incidence of PPCs may be partially attributed to differences in diagnostic criteria among studies. For example, some studies used strict radiological confirmation, while others relied on clinical symptoms combined with laboratory results. However, sensitivity analysis showed that the overall trend of IMT reducing PPCs remained consistent regardless of diagnostic criteria. Furthermore, in addition to its beneficial effects on the respiratory system, IMT also synergises with other perioperative management measures. For instance, by accelerating early postoperative mobilization, IMT strengthens respiratory muscles, enabling patients to tolerate deep breathing and active coughing within 24 hours post-surgery, reducing dependence on analgesics.

It is worth noting that heterogeneity was observed in key outcomes (e.g., FEV1, 6MWT), which may be attributed to several factors: (1) Differences in IMT protocols (e.g., resistance load, training duration, initiation timing); (2) Variations in patient characteristics (e.g., age, comorbidities such as COPD); (3) Differences in surgical types (before exclusion of cardiac surgery) and surgical approaches (open vs. minimally invasive). Sensitivity analysis excluding the outlier study (Hegazy FA et al.) reduced heterogeneity but did not change the overall conclusion, indicating the robustness of the results.

Therefore, we recommend the future widespread use of IMT in cardiothoracic surgery to counteract postoperative immobilization-induced muscle strength decline by enhancing respiratory muscle reserves. IMT should be particularly implemented for elderly patients, those with COPD comorbidities, or preoperative FEV1<60%, as these patients have poor respiratory muscle reserve function and derive greater benefit from IMT. Additionally, IMT should subsequently be combined with early mobilization, cough training, and nutritional support to form an ERAS closed-loop management system.

In view of the inclusion of both cardiac surgery (e.g., mitral valve replacement, coronary artery bypass grafting) and thoracic surgery (e.g., thoracoscopic lobectomy, esophagectomy) in this meta-analysis, it is necessary to discuss the potential differences in the impact of IMT between different surgical subtypes. Although these surgical subtypes differ in specific approaches (e.g., cardiothoracic surgery directly involves lung parenchyma, while cardiac surgery involves cardiopulmonary bypass), they all induce respiratory muscle dysfunction, reduced lung volume, and increased risk of PPCs due to intrathoracic manipulation, anesthesia, and pain-induced respiratory suppression (Ball et al., 2022; Fischer et al., 2022). Nevertheless, the heterogeneity observed in some outcomes (e.g., FEV1, 6MWT) may be partially attributed to the differences between cardiac and cardiothoracic surgery, as well as between open and minimally invasive approaches. Future studies may design stratified analyses based on surgical subtypes to further clarify the precise efficacy and optimal protocol of IMT in specific populations.

It is worth noting that this study still has several important limitations that need to be addressed, and the most prominent one is that the heterogeneity of surgical approaches, patient groups, IMT protocols, and outcome indicators has not been completely resolved. First, the included studies still cover different subtypes of cardiothoracic surgery (open cardiac surgery, thoracic resection, esophageal surgery), and the differences in respiratory impairment mechanisms lead to unavoidable clinical heterogeneity; in addition, there are significant variations in IMT protocols among studies, and the optimal dosage, initiation timing, and training duration of IMT in cardiothoracic surgery remain uncertain, which is the core factor affecting the homogeneity of the study results. Second, there are potential variations in routine perioperative care among different studies and medical centers: some centers integrate low-intensity respiratory muscle training into conventional nursing, which leads to the heterogeneity of control group interventions and may affect the evaluation of IMT efficacy. Third, the total sample size of the included studies is limited (only 507 cases); most studies focus on thoracoscopic lobectomy, with insufficient data on esophageal surgery patients, which limits the generalization of the conclusions to esophageal surgery populations. Fourth, only three studies reported postoperative PF data beyond one month, and no studies evaluated long-term quality of life or chronic respiratory function decline. This indicates that current evidence primarily supports the short-term physiological benefits of IMT (e.g., improved PF, reduced early PPCs), while its long-term functional outcomes and impact on quality of life remain uncertain. Fifth, most studies did not implement complete blinding for patients and researchers due to the characteristics of physical training interventions, which may introduce slight subjective bias (e.g., reduced compliance in the control group due to awareness of not receiving IMT); future RCTs need to optimize the blinding design (e.g., using sham training devices with the same appearance) to improve the level of evidence.

## Conclusion

Our systematic review and meta-analysis provide evidence supporting that IMT may improve respiratory muscle function and reduce the risk of short-term PPCs (e.g., pneumonia and atelectasis) in patients undergoing cardiothoracic surgery. However, these findings should be interpreted with caution due to inherent limitations of the included evidence: the heterogeneity of surgical approaches, patient groups, and IMT protocols has not been completely resolved, there are potential variations in routine nursing among different centers, and the optimal dosage, initiation timing, and training duration of IMT in cardiothoracic surgery remain uncertain. Future research should focus on the following aspects to further solve the existing problems and optimize the clinical application of IMT: first, design multicenter RCTs with homogeneous research populations to standardize IMT protocols and confirm the optimal training intensity, initiation timing, and duration for different cardiothoracic surgery subtypes; second, control the consistency of routine perioperative care among centers to reduce the heterogeneity of control group interventions; third, expand the sample size of esophageal surgery and other underrepresented surgical types to enhance the generalization of the conclusions; fourth, extend the follow-up period to 6~12 months to verify the long-term benefits of IMT on pulmonary function and quality of life; fifth, conduct stratified analyses based on surgical subtypes and patient characteristics to explore precise IMT intervention strategies for specific populations.

## Data Availability

The original contributions presented in the study are included in the article/supplementary material. Further inquiries can be directed to the corresponding author.

## References

[B1] Azambuja A. C. M. de Oliveira L. Z. Sbruzzi G. (2020). Inspiratory muscle training in patients with heart failure: What is new? Systematic review and meta-analysis. Phys. Ther. 100, 2099–2109. doi: 10.1093/ptj/pzaa171 32936904

[B2] Ball L. Volta C. A. Saglietti F. Spadaro S. Di Lullo A. De Simone G. . (2022). Associations between expiratory flow limitation and postoperative pulmonary complications in patients undergoing cardiac surgery. J. Cardiothorac. Vasc. Anesth. 36, 815–824. doi: 10.1053/j.jvca.2021.07.035 34404594

[B3] Benli R. K. Yurdalan U. Yilmaz B. Adiguzel N. (2024). Effect of post-extubation inspiratory muscle training on diaphragmatic function in mechanically ventilated patients: A randomized controlled trial. Adv. Clin. Exp. Med. 33, 1077–1085. doi: 10.17219/acem/174815 38230846

[B4] Bissett B. M. Leditschke I. A. Neeman T. Green M. Marzano V. Erwin K. . (2023). Does mechanical threshold inspiratory muscle training promote recovery and improve outcomes in patients who are ventilator-dependent in the intensive care unit? The IMPROVE randomised trial. Aust. Crit. Care 36, 613–621. doi: 10.1016/j.aucc.2022.07.002 36041982

[B5] da Silva Guimaraes B. de Souza L. C. Cordeiro H. F. Regis T. L. Leite C. A. Puga F. P. . (2021). Inspiratory muscle training with an electronic resistive loading device improves prolonged weaning outcomes in a randomized controlled trial. Crit. Care Med. 49, 589–597. doi: 10.1097/CCM.0000000000004787 33332819

[B6] Del Corral T. Fabero-Garrido R. Plaza-Manzano G. Fernandez-de-Las-Penas C. Navarro-Santana M. Lopez-de-Uralde-Villanueva I. (2023). Home-based respiratory muscle training on quality of life and exercise tolerance in long-term post-COVID-19: Randomized controlled trial. Ann. Phys. Rehabil. Med. 66, 101709. doi: 10.1016/j.rehab.2022.101709 36191860 PMC9708524

[B7] Ehrsam J. P. Aigner C. (2023). Surgery of old people-Thoracic surgery. Chirurgie (Heidelb). 94, 17–27. doi: 10.1007/s00104-022-01772-y 36441200 PMC9703435

[B8] Fernandez-Lazaro D. Gallego-Gallego D. Corchete L. A. Fernandez Zoppino D. Gonzalez-Bernal J. J. Garcia Gomez B. . (2021). Inspiratory muscle training program using the PowerBreath((R)): Does it have ergogenic potential for respiratory and/or athletic performance? A systematic review with meta-analysis. Int. J. Environ. Res. Public Health 18 (13), 6703. doi: 10.3390/ijerph18136703 34206354 PMC8297193

[B9] Fischer M. O. Brotons F. Briant A. R. Suehiro K. Gozdzik W. Sponholz C. . (2022). Postoperative pulmonary complications after cardiac surgery: The VENICE international cohort study. J. Cardiothorac. Vasc. Anesth. 36, 2344–2351. doi: 10.1053/j.jvca.2021.12.024 35094928

[B10] Hegazy F. A. Mohamed Kamel S. M. Abdelhamid A. S. Aboelnasr E. A. Elshazly M. Hassan A. M. (2021). Effect of postoperative high load long duration inspiratory muscle training on pulmonary function and functional capacity after mitral valve replacement surgery: A randomized controlled trial with follow-up. PloS One 16, e0256609. doi: 10.1371/journal.pone.0256609 34449776 PMC8396720

[B11] Jaenisch R. B. Silva C. C. F. Tonetto L. S. Gonzatti N. Guex C. G. Puntel G. O. . (2025). Respiratory muscle training attenuates oxidative stress and improves antioxidant activity in diabetic rats induced by high-fat diet and low-dose streptozotocin. Eur. J. Appl. Physiol. 126 (3), 1375–1389. doi: 10.1007/s00421-025-05956-2 40887537

[B12] Kulchanarat C. Choeirod S. Thadatheerapat S. Piathip D. Satdhabudha O. Yuenyongchaiwat K. (2025). Inspiratory muscle training improved cardiorespiratory performance in patients undergoing open heart surgery: A randomized controlled trial. Adv. Respir. Med. 93 (3), 10. doi: 10.3390/arm93030010 40558109 PMC12189406

[B13] Lin Y. Vervoort D. Thapa B. Sapkota R. Mitchell J. D. (2022). Minimally invasive thoracic surgery for low- and middle-income countries. Thorac. Surg. Clin. 32, 405–412. doi: 10.1016/j.thorsurg.2022.04.003 35961748

[B14] Litle V. R. (2023). Ever-evolving thoracic surgery: Chest is best. Thorac. Surg. Clin. 33, xi–xii. doi: 10.1016/j.thorsurg.2022.10.001 36372539

[B15] Liu J. F. Kuo N. Y. Fang T. P. Chen J. O. Lu H. I. Lin H. L. (2021). A six-week inspiratory muscle training and aerobic exercise improves respiratory muscle strength and exercise capacity in lung cancer patients after video-assisted thoracoscopic surgery: A randomized controlled trial. Clin. Rehabil. 35, 840–850. doi: 10.1177/0269215520980138 33307766

[B16] McNarry M. A. Berg R. M. G. Shelley J. Hudson J. Saynor Z. L. Duckers J. . (2022). Inspiratory muscle training enhances recovery post-COVID-19: a randomised controlled trial. Eur. Respir. J. 60 (4), 2103101. doi: 10.1183/13993003.03101-2021 35236727 PMC8900538

[B17] Mello E. S. F. Oliveira A. Santanna T. D. C. Soares P. Rodrigues G. D. (2024). Updates in inspiratory muscle training for older adults: A systematic review. Arch. Gerontol Geriatr. 127, 105579. doi: 10.1016/j.archger.2024.105579 39032314

[B18] Mizusawa H. Higashimoto Y. Shiraishi O. Shiraishi M. Sugiya R. Noguchi M. . (2025). Preoperative inspiratory muscle training preserved diaphragmatic excursion after esophagectomy: a randomized-controlled trial. Esophagus 22, 331–339. doi: 10.1007/s10388-025-01123-w 40178716 PMC12167333

[B19] Polastri M. Pehlivan E. Reed R. M. (2024). Inspiratory muscle training for lung transplant candidates and recipients. Exp. Clin. Transplant. 22, 479–486. doi: 10.6002/ect.2024.0073 39223806

[B20] Saka S. Gurses H. N. Bayram M. (2021). Effect of inspiratory muscle training on dyspnea-related kinesiophobia in chronic obstructive pulmonary disease: A randomized controlled trial. Complement. Ther. Clin. Pract. 44, 101418. doi: 10.1016/j.ctcp.2021.101418 34034036

[B21] Sakai Y. Yamaga T. Yamamoto S. Matsumori K. Ichiyama T. Hanaoka M. . (2023). Effects and usefulness of inspiratory muscle training load in patients with advanced lung cancer with dyspnea. J. Clin. Med. 12 (10), 3396. doi: 10.3390/jcm12103396 37240502 PMC10219417

[B22] Sorino C. Mondoni M. Lococo F. Marchetti G. Feller-Kopman D. (2022). Optimizing the management of complicated pleural effusion: From intrapleural agents to surgery. Respir. Med. 191, 106706. doi: 10.1016/j.rmed.2021.106706 34896966

[B23] Tao W. Huang J. Jin Y. Peng K. Zhou J. (2024). Effect of pulmonary rehabilitation exercise on lung volume and respiratory muscle recovery in lung cancer patients undergoing lobectomy. Altern. Ther. Health Med. 30, 90–96. 37883752

[B24] Van Hollebeke M. Poddighe D. Hoffman M. Clerckx B. Muller J. Louvaris Z. . (2025). Similar weaning success rate with high-intensity and sham inspiratory muscle training: A randomized controlled trial (IMweanT). Am. J. Respir. Crit. Care Med. 211, 381–390. doi: 10.1164/rccm.202405-1042OC 39565276

[B25] Xu L. Wei J. Liu J. Feng Y. Wang L. Wang S. . (2023). Inspiratory muscle training improves cardiopulmonary function in patients after transcatheter aortic valve replacement: a randomized clinical trial. Eur. J. Prev. Cardiol. 30, 191–202. doi: 10.1093/eurjpc/zwac269 36378543

[B26] Yu P. Luo Z. Wang Y. Lin S. Qin D. Jones A. Y. . (2025). Preoperative inspiratory muscle training improves lung function prior to elective heart valve surgery and reduces postoperative lung function impairment and pulmonary complications: a randomised trial. J. Physiother. 71, 27–34. doi: 10.1016/j.jphys.2024.12.002 39675947

[B27] Zhang Q. L. Ge M. Chen C. Fan F. D. Jin Y. Zhang N. . (2023). Comparison of effects of Liuzijue exercise and conventional respiratory training on patients after cardiac surgery: A randomized controlled trial. Chin. J. Integr. Med. 29, 579–589. doi: 10.1007/s11655-023-3637-9 37243804 PMC10224653

[B28] Zhou T. Sun C. (2022). Effect of physical manipulation pulmonary rehabilitation on lung cancer patients after thoracoscopic lobectomy. Thorac. Cancer. 13, 308–315. doi: 10.1111/1759-7714.14225 34882313 PMC8807280

