# Correction: The impact of inspiratory muscle training on pulmonary function recovery and pulmonary complications in patients undergoing cardiothoracic surgery: a systematic review and meta-analysis

**DOI:** 10.3389/fphys.2026.1889933

**Published:** 2026-06-05

**Authors:** Yinping Ge, Min Zhang, Yun Gan

**Affiliations:** 1 Department of Thoracic Surgery, Linping Campus, The Second Affiliated Hospital of Zhejiang University School of Medicine, Hangzhou, China; 2 Department of Neurosurgery, The Second Affiliated Hospital, Zhejiang University School of Medicine, Hangzhou, China; 3 Department of Infection Control, The Second Affiliated Hospital, Zhejiang University School of Medicine, Hangzhou, China

**Keywords:** cardiothoracic surgery, inspiratory muscle training, meta-analysis, postoperative pulmonary complications, pulmonary function

In the published article, there was an error in the affiliation of author “Yinping Ge.

Incorrect affiliation:

“Department of Thoracic Surgery, The Second Affiliated Hospital, Zhejiang University School of Medicine, Hangzhou, China”.

Correct affiliation:

“Department of Thoracic Surgery, Linping Campus, The Second Affiliated Hospital of Zhejiang University School of Medicine, Hangzhou, China”.

The original version of this article has been updated.

